# Supplementary material for: Two distinct ferredoxins are essential for nitrogen fixation by the iron nitrogenase in Rhodobacter capsulatus
Source: mBio. 2024 Feb 20;15(3):e03314-23. doi: 10.1128/mbio.03314-23 (PMC10936413; doi:10.1128/mbio.03314-23)
Supplement: Supplemental Material — Supplemental text, tables, and figures. [file mbio.03314-23-s0001.pdf]

Two distinct ferredoxins are essential for nitrogen fixation by the iron nitrogenase in *Rhodobacter capsulatus*

Holly Addison[1], Timo Glatter[2], Georg K. A. Hochberg[3, 4], Johannes G. Rebelein[1, 4]

1. Microbial Metalloenzymes Research Group, Max Planck Institute for Terrestrial Microbiology, 35043 Marburg, Germany.
2. Core Facility for Mass Spectrometry & Proteomics, Max Planck Institute for Terrestrial Microbiology, 35043 Marburg, Germany
3. Evolutionary Biochemistry Research Group, Max Planck Institute for Terrestrial Microbiology, 35043 Marburg, Germany.
4. Center for Synthetic Microbiology (SYNMIKRO), Philipps University Marburg, 35043 Marburg, Germany.

#Address correspondence: Johannes.Rebelein@mpi-marburg.mpg.de

Running title:
Ferredoxins driving the iron nitrogenase activity

Supplementary methods:

Proteome analysis

R. capsulatus strains were cultured anaerobically until a total OD_{660} of 3 was achieved. Cell samples were prepared by three centrifugation steps and two washing steps with phosphate buffer (3.6 g $Na_2HPO_4 \times 2 H_2O$ and 2.6 g KH_2PO_4 per litre distilled H_2O). For protein extraction frozen cell pellets were re-suspended in 2% sodium lauroyl sarcosinate (SLS) and heated for 15 min at 90°C. Proteins were reduced with 5 mM Tris(2-carboxyethyl) phosphine (Thermo Fischer Scientific) at 90°C for 15 min and alkylated using 10 mM iodoacetamid (Sigma Aldrich) at 20°C for 30 min in the dark. Proteins were precipitated with a 6-fold excess of ice cold acetone, followed by two methanol washing steps. Dried proteins were reconstituted in 0.2 % SLS and the amount of proteins was determined by bicinchoninic acid protein assay (Thermo Scientific). For tryptic digestion 50 µg protein was incubated in 0.5% SLS and 1 µg of trypsin (Serva) at 30°C overnight.

After digestion, SLS was precipitated by adding a final concentration of 1.5% trifluoroacetic acid (TFA, Thermo Fischer Scientific). Peptides were desalted by using C18 solid phase extraction cartridges (Macherey-Nagel). Cartridges were prepared by adding acetonitrile (ACN), followed by equilibration with 0.1% TFA. Peptides were loaded on equilibrated cartridges, washed with 5% ACN and 0.1% TFA containing buffer and finally eluted with 50% ACN and 0.1% TFA.

Peptides were dried and reconstituted in 0.1% trifluoroacetic acid and then analyzed using liquid-chromatography-mass spectrometry carried out on a Exploris 480 instrument connected to an Ultimate 3000 RSLC nano and a nanospray flex ion source (all Thermo Scientific). Peptide separation was performed on a reverse phase HPLC column (75 µm x 42 cm) packed in-house with C18 resin (2.4 µm; Dr. Maisch). The following separating gradient was used: 94% solvent A (0.15% formic acid) and 6% solvent B (99.85% acetonitrile, 0.15% formic acid) to 25% solvent B over 95 minutes at a flow rate of 300 nl/min, and an additional increase of solvent B to 35% for 25min. MS raw data was acquired in data independent acquisition mode with a method adopted from *Bekker-Jensen et al.*[1]. In short, Spray voltage were set to 2.3 kV, funnel RF level at 40, and heated capillary temperature at 275 °C. For DIA experiments full MS resolutions were set to 120.000 at m/z 200 and full MS, AGC (Automatic Gain Control) target was 300% with an IT of 50 ms. Mass range was set to 350–1400. AGC target value for fragment spectra was set at 3000%. 45 windows of 14 Da were used with an overlap of 1 Da. Resolution was set to 15,000 and IT to 22 ms. Stepped HCD collision energy of 25, 27.5, 30 % was used. MS1 data was acquired in profile, MS2 DIA data in centroid mode.

Analysis of DIA data was performed using DIA-NN version 1.8 using a UniProt protein database from *Rhodobacter capsulatus*[2]. Full tryptic digest was allowed with two missed cleavage sites, and oxidized methionines and carbamidomethylated cysteine. Match between runs and remove likely interferences were enabled. The neural network classifier was set to the single-pass mode, and protein inference was based on genes. Quantification strategy was set to any LC (high accuracy). Cross-run normalisation was set to RT-dependent. Library generation was set to smart profiling. DIA-NN outputs were further evaluated using the SafeQuant and script modified to process DIA-NN outputs[3, 4]. The SafeQuant script was executed on the “report.tsv” file from DIA-NN analysis to sum precursor intensities to represent protein intensities. The peptide-to-protein assignment was done in SafeQuant with redundant peptide assignment following the Occam´s razor approach. Median protein intensity normalization was performed followed by imputation of missing values using a normal distribution function. Log-ratio and significance value (Student´s t-Test) calculation was performed as a basis for Volcanoplots. P-values were calculated using eBayes moderated t-statistics[5].

Supplementary materials:

Table S1: Strains, plasmids and primers used in this study.

Strains:

Strain	Genotype	Reference
***R. capsulatus* strains**		
BS85 Δ*modABC*	Wild-type: Δ*nifD*::Sp mutant (Δ*nifDK*) Δ*modABC*	[6]
Δ*fdxB*	Δ*nifD*::Sp mutant (Δ*nifDK*) Δ*modABC* Δ*fdxB*	This study
Δ*fdxC*	Δ*nifD*::Sp mutant (Δ*nifDK*) Δ*modABC* Δ*fdxC*	This study
Δ*fdxD*	Δ*nifD*::Sp mutant (Δ*nifDK*) Δ*modABC* Δ*fdxD*	This study
Δ*fdxN*	Δ*nifD*::Sp mutant (Δ*nifDK*) Δ*modABC* Δ*fdxN*	This study
Δ*nifF*	Δ*nifD*::Sp mutant (Δ*nifDK*) Δ*modABC* Δ*nifF*	This study
Δ*fdxCN*	Δ*nifD*::Sp mutant (Δ*nifDK*) Δ*modABC* Δ*fdxCN*	This study
Δ*anfHGDK*	Δ*nifD*::Sp mutant (Δ*nifDK*) Δ*modABC* Δ*anfDGK*::*GmR*	This study
***E. coli* strains**		
DH5α	Φ80*lac*ZΔM15 Δ(*lac*ZYA-*arg*F) U169 *rec*A1 *end*A1 *hsd*R17(r_k^-, m_k^+) *pho*A *sup*E44 *thi*-1 *gyr*A96 *rel*A1 λ^-	Thermo Fisher Scientific Inc. (Waltham, USA) catalogue #18265017
ST18	RP4-2 *Tc*::*Mu Km*::*Tn7* Δ*hemA* mutant	[7]

Plasmids:

Plasmid	Relevant Features	Reference
pK18mob-SacB	Suicide plasmid, *sacB*, *oriT* (mobilisable), *lacZα* cassette, Km^R	[8]
pK18mob-SacB2	Suicide plasmid, *sacB*, *oriT* (mobilisable), *lacZα* cassette for golden gate cloning (BsaI), Km^R	This study
pK18mob-SacB2-Δ*fdxB*	Suicide plasmid, *sacB*, *oriT* (mobilisable), in-frame Δ*fdxB*, Km^R	This study
pK18mob-SacB2-Δ*fdxC*	Suicide plasmid, *sacB*, *oriT* (mobilisable), in-frame Δ*fdxC* into BsaI site of MCS, Km^R	This study
pK18mob-SacB2-Δ*fdxD*	Suicide plasmid, *sacB*, *oriT* (mobilisable), in-frame Δ*fdxD*, Km^R	This study
pK18mob-SacB2-Δ*fdxN*	Suicide plasmid, *sacB*, *oriT* (mobilisable), in-frame Δ*fdxN* into BsaI site of MCS, Km^R	This study
pK18mob-SacB2-Δ*nifF*	Suicide plasmid, *sacB*, *oriT* (mobilisable), in-frame Δ*nifF* into BsaI site of MCS, Km^R	This study
pK18mob-SacB2-Δ*fdxCN*	Suicide plasmid, *sacB*, *oriT* (mobilisable), in-frame Δ*fdxCN*, Km^R	This study
pOGG024-Km^R	Broad-host range plasmid, pBBR1 with *oriT* (mobilisable), *lacZα* cassette for golden gate cloning (BsaI), Km^R	[9]
pOGG024-2-Km^R	Broad-host range plasmid, pBBR1 with *oriT* (mobilisable), *lacZα* cassette for golden gate cloning (BsaI), AnfH promoter, Km^R	This study
pOGG024-2-Km^R *fdxN*	Broad-host range plasmid, pBBR1 with *oriT* (mobilisable), *fdxN* cloned into BsaI site, AnfH promoter, Km^R	This study
pOGG024-2-Km^R *fdxC*	Broad-host range plasmid, pBBR1 with *oriT* (mobilisable), *fdxC* cloned into BsaI site, AnfH promoter, Km^R	This study
pNMS16	Broad-host range plasmid, repABC4 with *oriT* (mobilisable), Km^R	This study
pNMS16-2	Broad-host range plasmid, repABC4 with *oriT* (mobilisable), *lacZα* cassette for golden gate cloning (BsaI), Km^R	This study

pNMS16-3	Broad-host range plasmid, repABC4 with *oriT* (mobilisable), AnfH promoter, Km^R	This study
pNMS16-3-*fdxN*	Broad-host range plasmid, repABC4 with *oriT* (mobilisable), *fdxN* cloned into MCS, AnfH promoter, Km^R	This study
pNMS16-3-*fdxC*	Broad-host range plasmid, repABC4 with *oriT* (mobilisable), *fdxC* cloned into MCS, AnfH promoter, Km^R	This study

Primers:

Primer	Sequence (5'-3')	Purpose
LacZ-F	TTACACTCTTCCGTAGGGGGTTACTCTAGGG	pOGG024 LacZ and MCS for Golden Gate into pNMS16 via Gibson
LacZ-R	GTCAAGTGGGATGCGTTCGGTCAAGGTTCTG	pOGG024 LacZ and MCS for Golden Gate into pNMS16 via Gibson
pK18mob-SacB-F	GACCGAACGCATCCCACTTGACCGAGATACA	Gibson cloning LacZ and MCS for Golden Gate into pNMS16
pK18mob-SacB-R	ACCCCCTACGGAAGAGTGTAAGCGGGTTGA	Gibson cloning LacZ and MCS for Golden Gate into pNMS16
anfHDGK-F	CACCACAGGTCTCGGGAGGCAGCCCGTTCGGAATTCC	GoldenGate anfH promoter into pNMS16-3
anfHGDK-R	CACCACAGGTCTCGAGCGTCACCACACGTTGAGGATCC	GoldenGate anfH promoter into pNMS16-3
anfHp-F	CAGCCCGTTCGGAATTCC	Gibson cloning anfH promoter into pOGG024-Km^R
anfHp-R	CATATGTATATCTCCTTCTTAAAGTTAAACAAAAT	Gibson cloning anfH promoter into pOGG024-Km^R
pOGG024-Km^R-F	CTTTAAGAAGGAGATATACATATGTGAGACCGCAGCTGGCACG	Gibson cloning anfH promoter into pOGG024-Km^R
pOGG024-Km^R-R	GGAATTCCGAACGGGCTGCAAATAAAACGAAAGGCTCAGTCG	Gibson cloning anfH promoter into pOGG024-Km^R
Δ*fdxB*-UF	GATTCAGGTCTCCCGGTCTGCTCGTCGATCTTCATCAGG	Amplify *fdxB* upstream homologous region from BS85
Δ*fdxB*-UR	GATCTAGGTCTCTTACGTCGGGCTTCTGGTCGAAAAG	Amplify *fdxB* upstream homologous region from BS85
Δ*fdxB*-DF	GATTCAGGTCTCCTTACATCAATCGGGGGCAAAGCAG	Amplify *fdxB* downstream homologous region from BS85
Δ*fdxB*-DR	GATTCAGGTCTCCACCGGGGCCGAGAATTGT	Amplify *fdxB* downstream homologous region from BS85
pK18mob-SacB-F	CGTAATAGCGAAGAGGCCCG	Amplification of pK18mob-SacB for Gibson cloning
pK18mob-SacB-R	GTAAAACGACGGCCAGTGC	Amplification of pK18mob-SacB for Gibson cloning
pK18mob-SacB-Qu-F	ACAATTCTCCTGCTCGTCGAT	Frame-shift correction via Quickchange in pK18mob-SacB-Δ*fdxB*

pK18mob-SacB-Qu-R	GGAGAATTGTATCGGCTGCG	Frame-shift correction via Quickchange in pK18mob-SacB-Δ*fdxB*
Δ*fdxC*-UF	GATCTAGGTCTCTATGGTTATCTGACCCATACGCGGC	Amplify *fdxC* upstream homologous region from BS85
Δ*fdxC*-UR	GATCTAGGTCTCCCTCGATGATGCGGGTTCC	Amplify *fdxC* upstream homologous region from BS85
Δ*fdxC*-DF	GATCTAGGTCTCTCGAGGAAATGCGGGTGCTGGAGG	Amplify *fdxC* downstream homologous region from BS85
Δ*fdxC*-DR	GATCTAGGTCTCCGGAGCCAGAAGATTGTTGCCCGAC	Amplify *fdxC* downstream homologous region from BS85
Δ*fdxD*-UF	GATCTAGGTCTCCAGGCCACCGCGTAGATGGTCTTGTC	Amplify *fdxD* upstream homologous region from BS85
Δ*fdxD*-UR	GATCTAGGTCTCCTACGGGTTGATCAGCGTCGACAGC	Amplify *fdxD* upstream homologous region from BS85
Δ*fdxD*-DF	GATCTAGGTCTCCTTACAGTCCACATCGTCATAGGCG	Amplify *fdxD* downstream homologous region from BS85
Δ*fdxD*-DR	GATCTAGGTCTCCGCCTGCCAGTTCATCCCG	Amplify *fdxD* downstream homologous region from BS85
Δ*fdxN*-UF	GATCTAGGTCTCTCGCACGCATTGGTCGGGCAGAC	Amplify *fdxN* upstream homologous region from BS85
Δ*fdxN*-UR	GATCTAGGTCTCTATGGCAGGGGCTGAAGATGCGTC	Amplify *fdxN* upstream homologous region from BS85
Δ*fdxN*-DF	GATCTAGGTCTCCGGAGATGCACGTCGAGCACCTG	Amplify *fdxN* downstream homologous region from BS85
Δ*fdxN*-DR	GATCTAGGTCTCCTGCGAAGGCGAGCATGAC	Amplify *fdxN* downstream homologous region from BS85
Δ*fdxCN*-UF	GATCTAGGTCTCCCTCGATGATGCGGGTTCC	Amplify *fdxCN* upstream homologous region from BS85
Δ*fdxCN*-UR	GATCTAGGTCTCTTACGGATCACCCCCTTCCTGGATC	Amplify *fdxCN* upstream homologous region from BS85
Δ*fdxCN*-DF	GATCTAGGTCTCCTTACATGCACGTCGAGCACCTG	Amplify *fdxCN* downstream homologous region from BS85
Δ*fdxCN*-DR	GATCTAGGTCTCCCGAGTGCGAAGGCGAGCATGAC	Amplify *fdxCN* downstream homologous region from BS85
pK18mob-SacB-F	GATTTAGGTCTCTGTAAAACGACGGCCAGTGC	GoldenGate cloning of *fdxD* or *fdxCN* homolgous regions into pK18mob-SacB

pK18mob-SacB-R	GATTTAGGTCTCTCGTAATAGCGAAGAGGCCCG	GoldenGate cloning of *fdxD* or *fdxCN* homolgous regions into pK18mob-SacB
Δ*nifF*-UF	GATCTAGGTCTCTATGGATATCAAAGGCCCCGGCG	Amplify *nifF* upstream homologous region from BS85
Δ*nifF*-UR	GATCTAGGTCTCTTGATCCTCATCGTCGAACATGTCC	Amplify *nifF* upstream homologous region from BS85
Δ*nifF*-DF	GATCTAGGTCTCTATCAGGACAATCAGGCGGC	Amplify *nifF* downstream homologous region from BS85
Δ*nifF*-DR	GATCTAGGTCTCTGGAGTGAAGGAATCCGACTGGTGC	Amplify *nifF* downstream homologous region from BS85
fdxN-Gi-F	CTTTAAGAAGGAGATATACATATGGCCATGAAGATCGATCCCGA	Amplify *fdxN* for Gibson cloning into pNMS16-3
fdxN-Gi-R	CAACAGGAGTCCAAGAGCGTTACGCCGCCGGGTTG	Amplify *fdxN* for Gibson cloning into pNMS16-3
fdxC-Gi-F	TTTACTTTAAGAAGGAGATATACATATGGACAAGGCCACACTGACGTT	Amplify *fdxC* for Gibson cloning into pNMS16-3
fdxC-Gi-R	TCAACAGGAGTCCAAGAGCGTCAGGCGGGGCGAACC	Amplify *fdxC* for Gibson cloning into pNMS16-3
pNMS16-3-F	CGCTCTTGGACTCCTGTTGA	Gibson cloning of *fdxN* or *fdxC* into pNMS16-3
pNMS16-3-R	CATATGTATATCTCCTTCTTAAAGTTAAACAAAAT	Gibson cloning of *fdxN* or *fdxC* into pNMS16-3
fdxN-Go-F	GATCTAGGTCTCTAGCGTTACGCCGCCGGGTTGATG	Amplify *fdxN* for GoldenGate into pOGG024-2-Km^R
fdxN-Go-R	GATCTAGGTCTCCTATGGCCATGAAGATCGATCCCGA	Amplify *fdxN* for GoldenGate into pOGG024-2-Km^R
fdxC-Go-F	GATCTAGGTCTCTAGCGTCAGGCGGGGCGAACCTT	Amplify *fdxC* for GoldenGate into pOGG024-2-Km^R
fdxC-Go-R	GATCTAGGTCTCCTATGATGGACAAGGCCACACTGACG	Amplify *fdxC* for GoldenGate into pOGG024-2-Km^R

Table S2: Whole proteome analysis of *R. capsulatus* grown under non-N_2-fixing conditions (NH_4^+) and N_2-fixing conditions (N-free). (Excel file attached).

Table S3: Log_2 ratios of N_2 fixation proteins between *R. capsulatus* grown under N_2-fixing conditions (N-free) and non-N_2-fixing conditions (NH_4^+).

**Protein code**	**Gene name**	**N-free.log_2ratio**	**-log_{10}_pValue.N-free**
D5ANJ7	*anfD*	12.59872	8.727144
D5ARX7	*fdxB*	12.27127	10.466
D5ANJ9	*anfK*	11.70715	7.152174
D5ANK2	*RCAP_rcc00591*	11.65023	5.812046

D5ANJ6	*anfH*	11.56207	6.997151
D5ANI3	*nifH*	11.27484	6.136328
D5ARX9	*RCAP_rcc03277*	10.85964	6.545178
D5ANI4	*fdxD*	9.679919	6.939873
D5ARW4	*RCAP_rcc03262*	8.987266	5.808905
D5ARY0	*nifX*	8.859228	3.927786
D5ARY7	*fdxC*	8.845235	6.675845
D5ANJ8	*anfG*	8.689296	8.207057
D5ARY1	*nifN*	8.256918	4.784761
D5ARW5	*RCAP_rcc03263*	8.068804	7.712417
D5ARZ3	*rnfG*	8.044382	9.263996
D5ARX3	*nifU2*	7.744715	6.812609
D5ARX8	*RCAP_rcc03276*	7.726194	5.40691
D5ARZ1	*rnfC*	7.536894	9.055173
D5ARY8	*fprA*	7.432053	8.5205
D5ARX4	*RCAP_rcc03272*	7.228431	6.836491
D5ARY2	*nifE*	7.128616	3.889748
D5ANK0	*anfO*	7.047755	4.065219
D5ARZ5	*rnfH*	7.017223	5.510083
D5ARX2	*nifS*	6.814573	3.258705
D5ANH9	*rpoN*	5.842829	5.168062
D5ARX0	*nifW*	5.350851	5.090256
D5ANJ5	*anfA*	4.307349	10.68301
D5ARY6	*fdxN*	3.79751	8.671147
D5ARY4	*rnfF*	3.636934	6.530411
D5ARZ0	*rnfB*	3.584759	4.715786
D5ARZ4	*rnfE*	2.869379	2.6354
D5ARZ2	*rnfD*	2.713729	3.150655
D5ANH7	*nifB1*	2.613498	5.460614
D5ANI2	*nifD*	2.559534	3.638374
D5APG0	*hupA*	2.451492	8.811083
D5ARY3	*RCAP_rcc03281*	2.308116	4.51212
D5APG1	*hupB*	2.262473	9.702227
P52967	*nifF*	1.856026	1.610574
P09431	*ntrB*	1.541781	8.008296
D5ANI6	*cowN*	1.529626	2.30679
P09432	*ntrC*	1.427897	8.858428
D5AKV9	*modA2*	1.408857	7.605173
D5ANH8	*nifA1*	1.288746	6.626321
D5ANH2	*mopA*	1.263358	7.549659
D5ANH6	*modD*	1.185609	8.222177

D5AKV8	*modB2*	0.857103	2.98255
D5ARX1	*nifV*	0.767107	0.711768
D5ANI1	*nifK*	0.235134	0.137075
D5AUA6	*ntrX*	0.165516	2.642961
D5AKW0	*modC2*	0.127946	0.454067
D5AUA5	*ntrY*	0.11615	0.570263
D5ARW3	*RCAP_rcc03261*	0.057427	0.651148
D5ARY9	*rnfA*	0.022949	0.207997
D5ANH1	*mopB*	-0.51132	6.632272
D5ANI0	*nifU1*	-1.23967	1.048143
D5AU34	*nifJ*	-2.28244	3.084097

All proteins with a log-fold-change of >±1 were considered up- or down-regulated between N_2-fixing conditions (WT (N-free)) and non-N_2 fixing conditions (WT (NH_4^+)). All proteins with a 0.01 P-value (log_{10}Adj.P-Value over 2.0) were considered significant. Boxes coloured in green are upregulated proteins, boxes in orange are downregulated proteins and boxes not coloured represent proteins that either are not up- or down- regulated or do not meet the 0.01 P-value cut-off. n/a stands for not applicable.

Table S4: Whole proteome analysis of WT, Δ*fdxN* and Δ*fdxC* *R. capsulatus* strains grown under N_2-fixing conditions. (Excel file attached).

Table S5: Log$_2$ ratios of N_2 fixation proteins between *R. capsulatus* Δ*fdxN* or Δ*fdxC* strains and the *R. capsulatus* WT.

Protein code	Gene name	Δ*fdxN*. log$_2$ratio	Δ*fdxC*. log$_2$ratio	-log$_{10}$_pValue. Δ*fdxN*	-log$_{10}$_pValue. Δ*fdxC*
D5ARZ0	*rnfB*	3.998439	3.027834	9.19467	9.239084
D5ARZ4	*rnfE*	3.885121	2.440782	7.046392	6.272526
D5ARZ5	*rnfH*	3.87851	3.116919	8.86022	9.117162
D5ARZ1	*rnfC*	3.81805	2.007708	7.632947	6.38165
D5ARY9	*rnfA*	3.668276	2.692474	5.858928	6.280726
D5ARZ2	*rnfD*	3.451044	2.02617	6.755926	5.853414
D5ARZ3	*rnfG*	3.156771	3.933726	11.36347	11.94346
P52967	*nifF*	2.90767	3.871691	3.493402	4.201193
D5ARW4	*RCAP_rcc03262*	2.102174	2.491553	8.052484	8.983435
D5ANI2	*nifD*	2.049606	2.697424	6.784045	8.173014
D5ARY7	*fdxC*	1.949947	n/a	7.054317	9.579414
D5ARX4	*RCAP_rcc03272*	1.839633	2.043956	6.623682	8.086195
D5ARY4	*rnfF*	1.827542	2.151687	6.008059	7.963533
D5ARX9	*RCAP_rcc03277*	1.810358	2.6456	6.698211	8.144624

D5ARX8	*RCAP_rcc03276*	1.781268	2.718973	7.896731	9.972142
D5ARX7	*fdxB*	1.730894	2.338911	7.390378	5.618482
D5ARY8	*fprA*	1.698857	1.390543	9.248192	8.597107
D5ANH7	*nifB1*	1.667965	2.165186	5.687121	6.819651
D5ANJ8	*anfG*	1.658196	1.327339	4.931956	5.164582
D5ARW5	*RCAP_rcc03263*	1.561616	2.14193	6.018241	6.937882
D5ARX0	*nifW*	1.544861	1.776224	5.661919	6.866204
D5ANJ6	*anfH*	1.51508	1.553795	5.930304	6.645611
D5ANI3	*nifH*	1.499192	2.024093	5.430483	6.650974
D5ANH9	*rpoN*	1.461493	1.81334	7.179225	7.045798
D5ANK0	*anfO*	1.456562	1.117837	4.504814	5.400112
D5ANJ7	*anfD*	1.451124	1.070575	5.183646	5.506334
D5ARX3	*nifU2*	1.334243	2.048841	6.343032	8.711973
D5ANI5	*RCAP_rcc00574*	1.26887	-1.97086	2.555368	1.494688
D5ANJ9	*anfK*	1.265425	0.462979	6.1094	4.527944
D5ANK2	*RCAP_rcc00591*	1.228654	0.917518	5.132341	5.127066
D5ARY2	*nifE*	1.181117	1.426355	5.994891	6.197274
D5ARY0	*nifX*	1.165747	1.830177	5.883956	8.474927
D5ARX2	*nifS*	1.128982	1.373777	4.826705	5.090009
D5ARY1	*nifN*	1.092217	1.479451	7.018562	8.523274
D5ANI4	*fdxD*	1.045993	2.66351	5.026649	10.22016
D5AKV8	*modB2*	0.949781	-0.13845	2.719426	0.608573
D5AU34	*nifJ*	0.932884	0.563581	1.56201	0.062862
D5ARX1	*nifV*	0.900374	0.892005	1.421565	1.07132
D5ANJ5	*anfA*	0.845423	0.454307	5.526384	3.873301
D5ARY3	*RCAP_rcc03281*	0.766416	-0.21199	1.145381	0.291941
D5AKW0	*modC2*	0.725204	-0.67275	3.229507	4.379903
D5ARW3	*RCAP_rcc03261*	0.622173	1.069767	4.260913	7.233544
D5ANI6	*cowN*	0.604531	-2.81857	0.87265	4.595866
D5ANI1	*nifK*	0.472799	1.226359	0.550185	1.28607
D5ANI0	*nifU1*	0.387758	3.356679	0.548121	3.347977
D5AKV9	*modA2*	0.359114	0.167948	2.200597	1.790392
D5AUA5	*ntrY*	0.170983	-0.17455	0.712654	0.738389
D5AUA6	*ntrX*	-0.01533	-0.02527	0.126457	0.241044
D5ANH2	*mopA*	-0.14889	-0.55089	1.457109	5.424188
D5ASC6	*mod*	-0.15287	-0.17444	2.307833	2.492609
D5ANH8	*nifA1*	-0.16688	-1.12742	1.013266	6.500072
D5ANH1	*mopB*	-0.19284	-0.88574	2.346441	6.935616

P09431	*ntrB*	-0.20457	0.399407	3.251092	3.810276
P09432	*ntrC*	-0.22451	-0.30686	3.136265	3.8755
D5ANH6	*modD*	-0.27382	-0.19504	3.625535	2.871633
D5APG0	*hupA*	-0.71449	-0.51455	5.927237	5.191983
D5APG1	*hupB*	-0.72956	-0.61754	6.664283	6.304395
D5ARY6	*fdxN*	n/a	5.193566	2.473119	2.53946

All proteins with a log-fold-change of >±1 were considered up- or downregulated between ∆*fdxN* or ∆*fdxC* strains (∆*fdxN* or ∆*fdxC*) and the WT (WT). All proteins with a 0.01 P-value (log_{10}Adj.P-Value over 2.0) were considered significant. All strains were grown diazotrophically in RCV minimal medium under an N_2 atmosphere (N_2-fixing conditions). Boxes coloured in green are upregulated proteins, boxes in orange are downregulated proteins and boxes not coloured represent proteins that either are not up- or down- regulated or do not meet the 0.01 P-value cut-off. n/a stands for not applicable.

Table S6. Sequences used for phylogenetic reconstructions of ferredoxins from *Rhodobacter capsulatus*.

Protein	Sequence (FASTA)
FdA	>WP_013068498.1 ferredoxin family protein [Rhodobacter capsulatus]<br>MTYVVTDNCIACKYTDCVEVCPVDCFYEGENTLVIHPDECIDCGVCE<br>PECPADAIRPDTEPGMEDWVEFNRTYASQWPVITIKKDPMPDHKKY<br>DGETGKREKYFSPNPGTGD
FdB	>WP_013068971.1 ferredoxin III, nif-specific [Rhodobacter capsulatus]<br>MMPTVAYTRGGAEYTPVYLMKIDEQKCIGCGRCFKVCGRDVMSLHG<br>LTEDGQVVAPGTDEWDEVEDEIVKKVMALTGAENCIGCGACARVCP<br>SECQTHAALS
FdN	>WP_013068980.1 ferredoxin FdxN [Rhodobacter capsulatus]<br>MAMKIDPELCTSCGDCEPVCPTNAIAPKKGVYVINADTCTECEGEH<br>DLPQCVNACMTDNCINPAA
FdC	>WP_013068981.1 ferredoxin FdxC [Rhodobacter capsulatus]<br>MDKATLTFTDVSITVNVPTGTRIIEMSEKVGSGITYGCREGECGTCM<br>THILEGSENLSEPTALEMRVLEENLGGKDDRLACQCRVLGGAVKVRPA
FdD	>WP_013066317.1:1-123 ferredoxin FdxD [Rhodobacter capsulatus]<br>MPNITFTSPIMKKDKTIYAVAGNTATILALAKEHAIPIPFECGDGDCAS<br>CLIEVTHLDNKPAMAMMLTEKEKARLKELQMITAEEIEAAEVSDLPP<br>RFRLACQFIPRDEDVMVHFTGTPGGSV
FdE	>WP_013068113.1:1-107 ferredoxin FdxE [Rhodobacter capsulatus]<br>MAKIIFIEHNGTRHEVEAKPGLTVMEAARDNGVPGIDADCGGACACS<br>TCHAYVDPAWVDKLPKALPTETDMIDFAYEPNPATSRLTCQIKVTSL<br>LDGLVVHLPEKQI

Sequences were retrieved from the UniProt database.

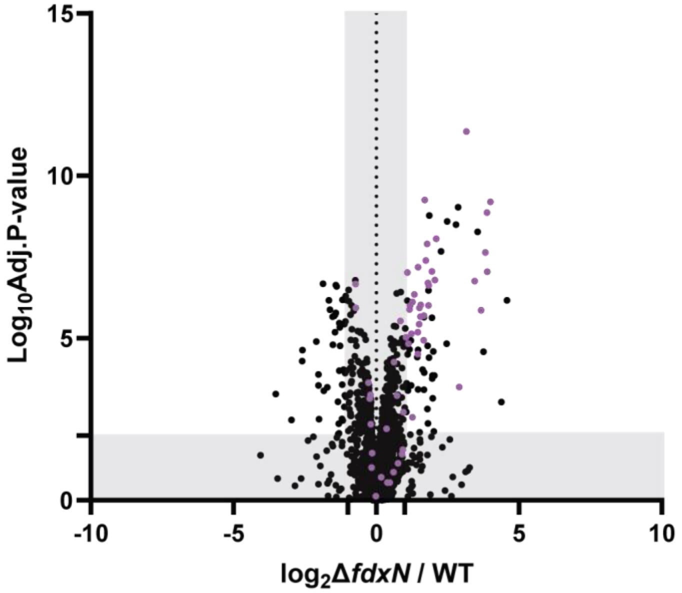

Fig S1. Up-regulation of nitrogen fixation-related proteins in *R. capsulatus* Δ*fdxN* strain relative to *R. capsulatus* WT. Volcano plot displaying mean intensity log_2-ratios of proteins (X-axis) versus significance values (-log_{10} adjusted P-values, Y-axis) between *R. capsulatus* Δ*fdxN* strains and *R. capsulatus* WT. N_2 fixation-related proteins are shown in purple. All proteins with a log_2-fold-change of >±1 were considered up- or down-regulated between *R. capsulatus* Δ*fdxN* and *R. capsulatus* WT strains. All proteins with a 0.01 P-value (log_{10}Adj.P-Value over 2.0) were considered significant. Grey boxes highlight regions of log_2fold values of -1> n <1 and log_{10}P-values of <2. Coefficients of variation between 4 independent cultures provided in Table S4. All strains carry an in-frame deletion of Δ*nifD* Δ*modABC* to ensure the expression of Fe-nitrogenase genes (*anf*). *R. capsulatus* strains were grown diazotrophically in RCV minimal medium under an N_2 atmosphere.

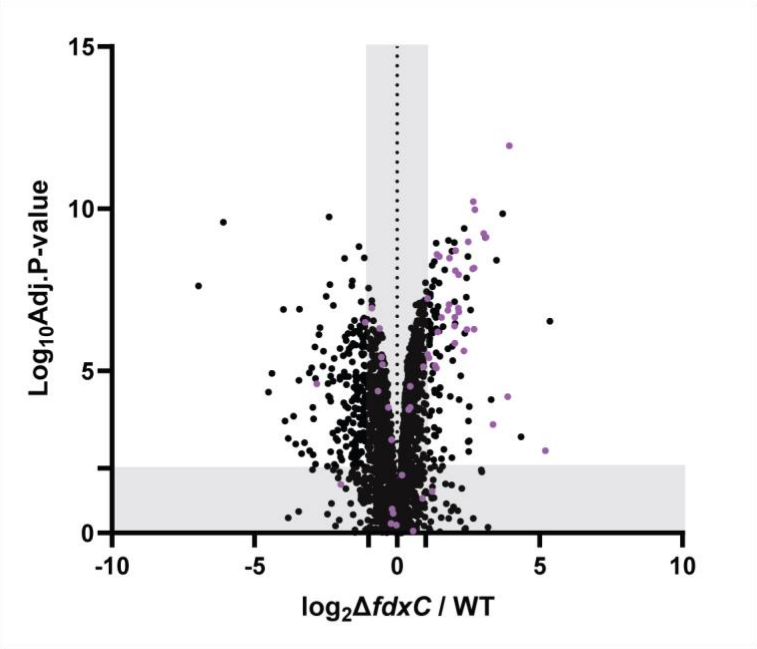

Fig. S2. Up-regulation of nitrogen fixation-related proteins in *R. capsulatus* ∆*fdxC* strain relative to *R. capsulatus* WT. Volcano plot displaying mean intensity log_2-ratios of proteins (X-axis) versus significance values (-log_{10} adjusted P-values, Y-axis) between *R. capsulatus* ∆*fdxC* strains and *R. capsulatus* WT. N_2 fixation-related proteins are shown in purple. All proteins with a log_2-fold-change of >±1 were considered up- or down-regulated between *R. capsulatus* ∆*fdxC* and *R. capsulatus* WT strains. All proteins with a 0.01 P-value (log_{10}Adj.P-Value over 2.0) were considered significant. Grey boxes highlight regions of log_2fold values of -1> n <1 and log_{10}P-values of <2. Coefficients of variation between 4 independent cultures provided in Table S4. All strains carry an in-frame deletion of ∆*nifD* ∆*modABC* to ensure the expression of Fe-nitrogenase genes (*anf*). *R. capsulatus* strains were grown diazotrophically in RCV minimal medium under an N_2 atmosphere.

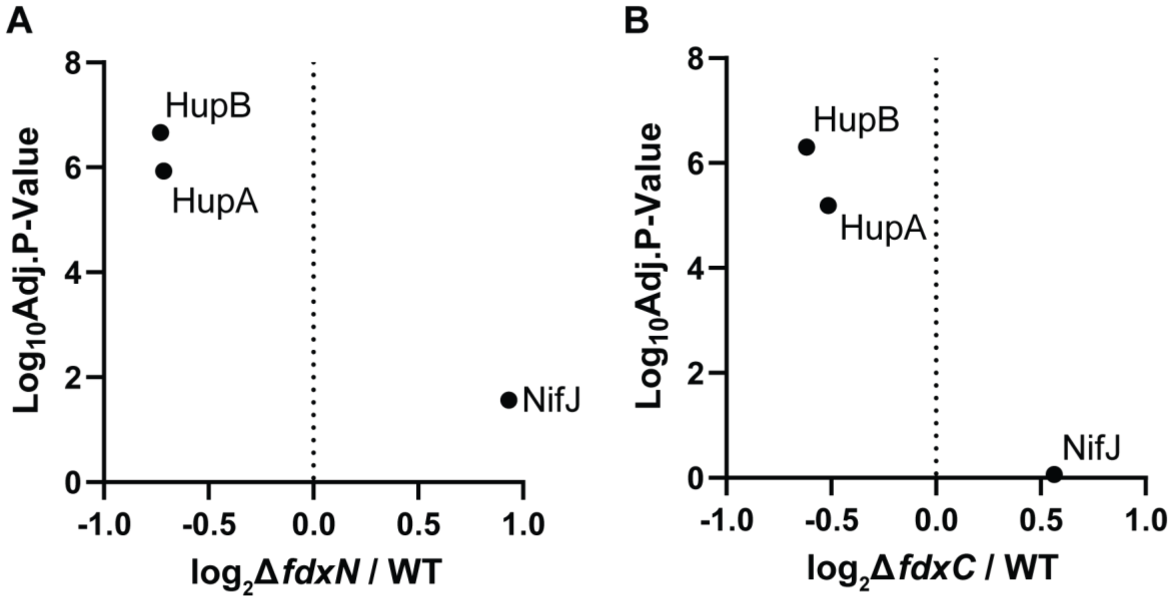

Fig. S3. No significant abundance changes in NifJ, HupA and HupB in ∆*fdxN* and ∆*fdxC* vs WT. (A) Scatter plot displaying mean intensity log_2-ratios (X-axis) versus significance values (-log_{10} adjusted P-values, Y-axis) of NifJ, HupA and HupB in ∆*fdxN* compared to the WT. **(B)** Scatter plot displaying mean intensity log_2-ratios (X-axis) versus significance values (-log_{10} adjusted P-values, Y-axis) of NifJ, HupA and HupB in ∆*fdxC* to compared to the WT. (A-B) All strains carry an in-frame deletion of ∆*nifD* ∆*modABC* to ensure the expression of Fe-nitrogenase genes (*Anf*). *R. capsulatus* strains were grown diazotrophically in RCV minimal medium under an N_2 atmosphere. All proteins with a 0.01 P-value (log_{10}Adj.P-Value over 2.0) were considered significant. All proteins with a log_2-fold-change of value of >±1 were considered over or under produced, between ∆*fdxN* or ∆*fdxC* to WT. Coefficients of variation between 4 independent cultures provided in Table S4.

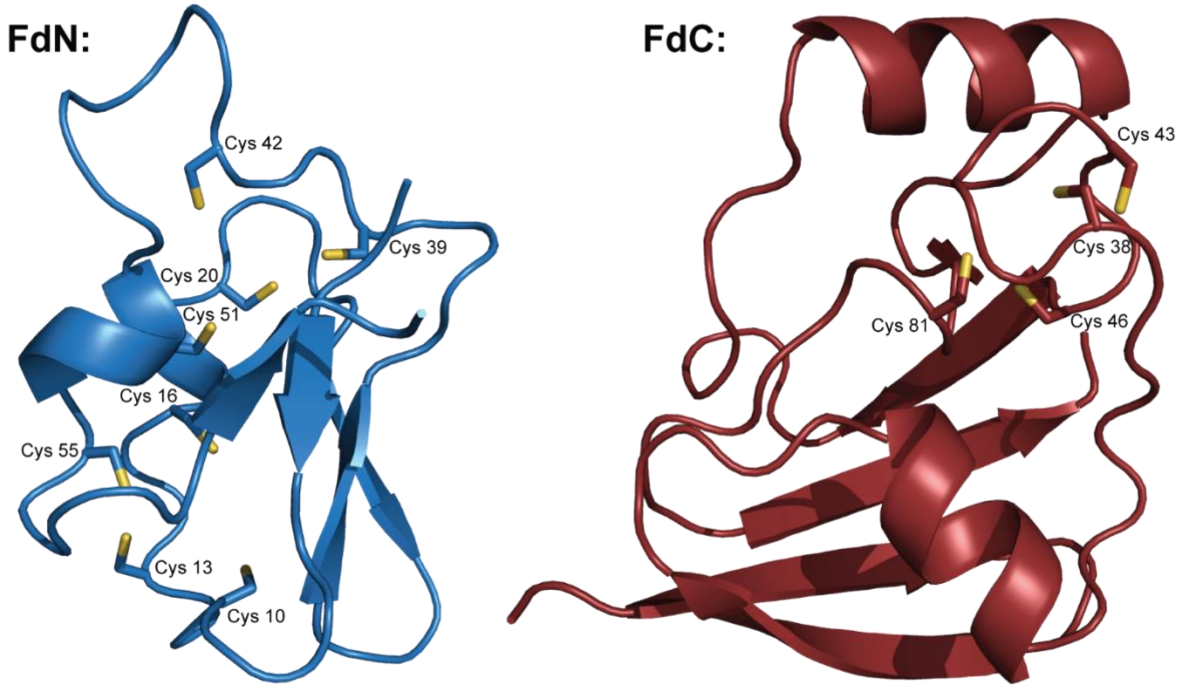

Fig. S4. AlphaFold predicted structures of FdN and FdC from *R. capsulatus*. AlphaFold models of FdN (left, blue) and FdC (right, red) retrieved from UniProt[10, 11]. Models are ribbon presentations of FdN and FdC with FeS cluster-coordinating cysteines labelled and shown as stick models with sulphur in yellow. UniProt identifier codes and AlphaFold structure identifier codes are: for FdN: D5ARY6, AF-D5ARY6-F1 and for FdC: D5ARY7, AF-D5ARY7-F1.

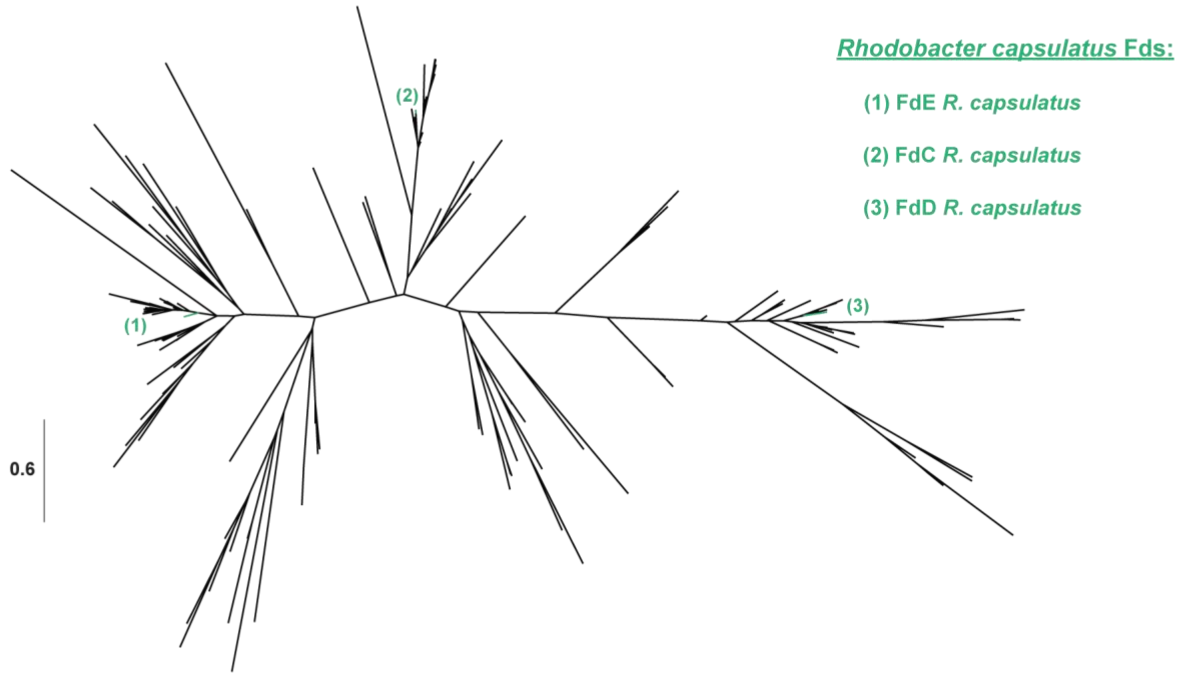

Fig. S5. Unrooted phylogenetic tree of [Fe_2S_2]-cluster containing ferredoxins from *R. capsulatus*. *R. capsulatus* Fd sequences are coloured in green and numbered as according to the green key on the right.

References:

1 Bekker-Jensen, D. B., Martínez-Val, A., Steigerwald, S., Rüther, P., Fort, K. L., Arrey, T. N., Harder, A., Makarov, A. and Olsen, J. V. (2020) A Compact Quadrupole-Orbitrap Mass Spectrometer with FAIMS Interface Improves Proteome Coverage in Short LC Gradients. Mol Cell Proteomics. **19**, 716-729

2 Demichev, V., Messner, C. B., Vernardis, S. I., Lilley, K. S. and Ralser, M. (2020) DIA-NN: neural networks and interference correction enable deep proteome coverage in high throughput. Nat Methods. **17**, 41-44

3 Glatter, T., Ludwig, C., Ahrné, E., Aebersold, R., Heck, A. J. R. and Schmidt, A. (2012) Large-Scale Quantitative Assessment of Different In-Solution Protein Digestion Protocols Reveals Superior Cleavage Efficiency of Tandem Lys-C/Trypsin Proteolysis over Trypsin Digestion. Journal of Proteome Research. **11**, 5145-5156

4 Ahrné, E., Molzahn, L., Glatter, T. and Schmidt, A. (2013) Critical assessment of proteome-wide label-free absolute abundance estimation strategies. PROTEOMICS. **13**, 2567-2578

5 Kammers, K., Cole, R. N., Tiengwe, C. and Ruczinski, I. (2015) Detecting significant changes in protein abundance. EuPA Open Proteomics. **7**, 11-19

6 Demtröder, L., Pfänder, Y. and Masepohl, B. (2020) Rhodobacter capsulatus AnfA is essential for production of Fe-nitrogenase proteins but dispensable for cofactor biosynthesis and electron supply. Microbiologyopen. **9**, 1234-1246

7 Thoma, S. and Schobert, M. (2009) An improved Escherichia coli donor strain for diparental mating. FEMS Microbiology Letters. **294**, 127-132

8 Schäfer, A., Tauch, A., Jäger, W., Kalinowski, J., Thierbach, G. and Pühler, A. (1994) Small mobilizable multi-purpose cloning vectors derived from the Escherichia coli plasmids pK18 and pK19: selection of defined deletions in the chromosome of Corynebacterium glutamicum. Gene. **145**, 69-73

9 Schmidt, F. V., Schulz, L., Zarzycki, J., Oehlmann, N. N., Prinz, S., Erb, T. J. and Rebelein, J. G. (2023) Structural Insights into the Iron Nitrogenase Complex. Nature Structural & Molecular Biology, 10.1038/s41594-41023-01124-41592

10 Jumper, J., Evans, R., Pritzel, A., Green, T., Figurnov, M., Ronneberger, O., Tunyasuvunakool, K., Bates, R., Žídek, A., Potapenko, A., Bridgland, A., Meyer, C., Kohl, S. A. A., Ballard, A. J., Cowie, A., Romera-Paredes, B., Nikolov, S., Jain, R., Adler, J., Back, T., Petersen, S., Reiman, D., Clancy, E., Zielinski, M., Steinegger, M., Pacholska, M., Berghammer, T., Bodenstein, S., Silver, D., Vinyals, O., Senior, A. W., Kavukcuoglu, K., Kohli, P. and Hassabis, D. (2021) Highly accurate protein structure prediction with AlphaFold. Nature. **596**, 583-589

11 Varadi, M., Anyango, S., Deshpande, M., Nair, S., Natassia, C., Yordanova, G., Yuan, D., Stroe, O., Wood, G., Laydon, A., Žídek, A., Green, T., Tunyasuvunakool, K., Petersen, S., Jumper, J., Clancy, E., Green, R., Vora, A., Lutfi, M., Figurnov, M., Cowie, A., Hobbs, N., Kohli, P., Kleywegt, G., Birney, E., Hassabis, D. and Velankar, S. (2022) AlphaFold Protein Structure Database: massively expanding the structural coverage of protein-sequence space with high-accuracy models. Nucleic Acids Res. **50**, D439-D444
